# Oral health of an indigenous population in northeastern Brazil: a cross-sectional Study of the Fulni-ô ethnic group

**DOI:** 10.1590/1516-3180.2022.0355.R1.10042023

**Published:** 2023-07-31

**Authors:** Bruna Del Vechio Koike, Rosangela Maria Pereira Valões, Claudia Cazal, Vanessa Cardoso Pereira, Carlos Alberto de Carvalho Fraga, Rodrigo Feliciano do Carmo, Meireane Firmino Pereira, Manoel Pereira Guimarães, Carlos Dornels Freire de Souza, Anderson da Costa Armstrong

**Affiliations:** I PhD. Biomedical and Adjunct Professor, Medical Course, College of Medicine, Universidade Federal do Vale do São Francisco (UNIVASF), Petrolina (PE), Brazil.; II MSc. Dental Surgeon, Department of Family Health Strategy, Municipality of Mirandiba, Mirandiba (PE), Brazil.; III PhD. Dentist and Adjunct Professor, Department of Odontology, College of Odontology, Universidade Federal de Pernambuco (UFPE), Recife (PE), Brazil.; IV MSc. Nurse, Department of Health Strategy, Municipality of Petrolina, Petrolina (PE), Brazil.; V PhD. Biologist and Adjunct Professor, Medical Course, College of Medicine, Universidade Federal de Alagoas (UFAL), Arapiraca (AL), Brazil.; VI PhD. Biologist and Adjunct Professor, Department of Pharmacy, College of Pharmacy, Universidade Federal do Vale do São Francisco (UNIVASF), Petrolina (PE), Brazil.; VII Dentist and Master’s Student, Universidade Federal de Alagoas (UFAL), Arapiraca (AL), Brazil.; VIII Undergraduate Student, College of Medicine, Universidade Federal do Vale do São Francisco (UNIVASF), Petrolina (PE), Brazil.; IX PhD, Epidemiologist and Adjunct Professor, Medical Course, College of Medicine, Universidade Federal do Vale do São Francisco (UNIVASF), Petrolina (PE), Brazil.; X PhD, Adjunct Professor, Department of Medicine, College of Medicine, Universidade Federal do Vale do São Francisco (UNIVASF), Petrolina (PE), Brazil.

**Keywords:** Oral health, Tobacco use disorder, Epidemiology, Indigenous peoples, Pathology, Health of indigenous peoples, Cross-sectional study, Health, public, Oral epithelial dysplasia, Oral cavity

## Abstract

**BACKGROUND:**

There is a lack of studies evaluating the oral health of traditional indigenous communities in Brazil.

**OBJECTIVES:**

Thus, the objective of this study was to describe the oral health characteristics of the indigenous Fulni-ô ethnic group in Northeast Brazil.

**DESIGN AND SETTING:**

A cross-sectional observational investigation was conducted within the Project on Atherosclerosis among Indigenous Populations.

**METHODS:**

This study included participants of both sexes from the Fulni-ô ethnic group. The participants included in this investigation underwent a comprehensive oral health evaluation by a registered and experienced dentist to assess oral health and identify potentially malignant oral lesions. Participants with suspicious lesions were referred for biopsy. Shapiro-Wilk, Mann-Whitney, and Student’s t-tests were used, and measures of central tendency and dispersion were described. Statistical significance was 5%.

**RESULTS:**

A total of 104 individuals were included in this study. The prevalence of the use of tobacco derivatives was 94.0%, with similarities between sexes. The prevalence of oral changes in this study population was 84.4%. Fifty-one individuals who underwent oral reassessment were referred for oral lesion biopsy.

**CONCLUSIONS:**

This study demonstrated a high prevalence of oral alterations in the Fulni-ô population. Histopathological analyses indicated the presence of mild oral epithelial dysplasia in five cases.

## INTRODUCTION

In 2003, with the discussions around the implementation of the National Oral Health Policy (Política Nacional de Saúde Bucal [PNSB]), called “Smiling Brazil,” Brazil took an important step in the process of building the integrality in health, considering the importance of the policy for the provision of free dental care within the Unified Health System (Sistema Único de Saúde [SUS]).^1,2^ Over the following years, the PNSB sought to reorganize primary care in oral health (especially with the implementation of Oral Health teams in the Family Health Strategy), the expansion and qualification of specialized care (especially with the implementation of Dental Specialty Centers and Regional Dental Prosthesis Laboratories), and the feasibility of adding fluoride to public water treatment plants.^3^

Between 2003 and 2014, federal funding for actions aimed at dental care for the Brazilian population increased from R$83.4 million to approximately R$916 million, respectively.^2^ Oral health surveys conducted in 2003 and 2010 showed a positive impact of the PNSB in reducing the frequency of untreated caries, periodontal damage, and tooth loss.^4,5^ However, there is still a long way to go in the construction of oral health practices capable of reaching all different Brazilian social conjunctures, marked by regional epidemiological and socioeconomic disparities.^4,5^ These disparities are more accentuated when discussing the access of indigenous populations to SUS, given their sociocultural and historical particularities.^6–9^

The Indigenous people of Brazil live across the country and have different ways of life. A total of 817,963 people declared themselves indigenous in the last demographic census in Brazil.^10^ They are distributed in more than 305 ethnicities along the Brazilian territory.^11^ One of the most traditional tribes in northeastern Brazil, and less urbanized, is the Fulni-ô.^12^ They live on San Francisco Valley region, in the northeast of Brazil, and they are the only tribe in the region that maintain their own language (Yathê) to date, keeping local traditions.^13^

Even though the first epidemiological studies on the oral health conditions of Brazilian indigenous populations began in the 1950s,^14^ little progress has been made in the production of knowledge on this subject in Brazil.

## OBJECTIVE

Thus, this study aimed to describe the oral health characteristics of an indigenous Fulni-ô ethnic group in the municipality of Águas Belas, Pernambuco State, Brazil.

## METHODS

### Study design, population, and period

This cross-sectional observational investigation is a continuation of the Project of Atherosclerosis Among Indigenous Populations (PAI). The PAI study has been described in previous studies.^15^ In summary, PAI is an observational study elaborated to access cardiovascular health in indigenous communities and has evaluated more than a thousand participants. During the study period, individuals of the Fulni-ô ethnic group frequently smoked and had compromised oral health.

The present investigation included participants of Fulni-ô ethnicity of both sexes aged 30 years or older. A non-probability sample was adopted, with the inclusion of all individuals who presented on the date of the oral health evaluation. Data was collected in the community.

The exclusion criteria were individuals with clinical heart failure, past acute coronary events that resulted in hospitalization, renal failure or dialysis, surgical history of cardiac or peripheral arterial procedure, or cerebrovascular disease that required hospitalization. These criteria are part of the PAI studies.

**Figure 1:**
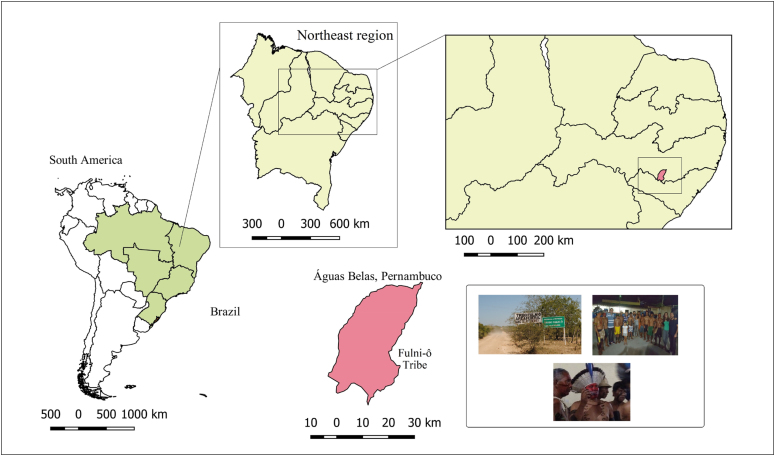
Study area. Fulni-ô tribe, Águas Bela, Pernambuco, Brazil.

The Fulni-ô people are considered to have a low level of urbanization. The Fulni-ô tribe is located on the banks of the Ipanema River (Águas Belas, state of Pernambuco, Brazil), a tributary of the São Francisco River (Figure 1).

### Variables

We analyzed sociodemographic variables (sex, age, and education) and lifestyle habits (alcohol consumption, use of tobacco products, use of commercial cigarettes, use of traditional herbal pipe – Xanduca smoking, habits of inhaling or chewing tobacco, cardiovascular complaints, and presence of comorbidities). The following cardiovascular complaints were observed: chest pain, spontaneous dyspnea, claudication associated with peripheral arterial disease, history of infarction, myocardial revascularization, and stroke.

Moreover, regarding the use of Xanduca, the average daily consumption (in units), the time of consumption (in years), and the consumption load (years x units consumed) were evaluated. Xanduca is produced from natural herbs in the region, and its consumption is related to its cosmology as *a particular rite*. Women believe that smoking Xanduca while performing prayers facilitates childbirth, and its consumption is recommended to prevent maternal mortality and protect pregnancy.^13^

Regarding oral health, the presence of oral cavity lesions and characteristics were evaluated.

### Oral lesions assessment

The participants included in this investigation underwent a comprehensive oral health assessment performed by a registered and experienced odontologist to evaluate their oral health and identify potentially malignant oral lesions. Patients with suspected lesions were referred for a biopsy. Adequate immediate treatment was provided to all patients, if suitable.

When necessary, a biopsy was performed using an incisional technique with previous asepsis of the lesion and local anesthesia. The specimens were fixed in formalin solution, individually identified, and sent for histopathological analysis.

### Histopathological procedures

Specimens were embedded in paraffin and stained with hematoxylin and eosin. Microscopic examination of sections revealed areas of epithelial dysplasia. Oral epithelial dysplasia was graded as mild, moderate, or severe based on the World Health Organization (WHO) criteria:^16^ whether dysplastic features were restricted to the lower third (mild dysplasia), middle third (moderate dysplasia), or upper third of the epithelium (severe dysplasia). Carcinoma *in situ* is used synonymously for severe dysplasia. All oral lesions found were then classified as benign, premalignant, or malignant, according to the final recommendations of the International Agency for Research on Cancer.^16^

### Statistical analysis

Statistical analyses were performed after collecting and structuring databases. Initially, the Shapiro-Wilk test was applied to assess data normality. In the descriptive analysis, measures of central tendency and dispersion (mean and standard deviation) were used for continuous variables and absolute and relative frequencies for categorical variables. In analytical statistics, the following tests were used: Mann-Whitney or Student t-tests for comparison of continuous variables between two groups as indicated and χ^2^ for association between qualitative variables. A level of 5% was considered statistically significant. JASP software was used for the analyses (Jeffreys’ Amazing Statistics Program, version 0.16.1, Department of Psychological Methods, University of Amsterdam, Amsterdam, The Netherlands).

### Ethical aspects

This study was approved by the Brazilian National Commission for Ethics in Research (CONEP) (number 48235615.9.0000.5196, April 13, 2016), the National Indigenous Foundation (Fundação Nacional dos Povos Indígenas [FUNAI]; process number 08620.028965/2015-66), and the indigenous leaders of the participating groups. All the participants provided written informed consent.

## RESULTS

A total of 104 individuals were included in the study, 66 (63.5%) were female with a mean age of 54 ± 11.5 years (34–96 years), with no difference between the sexes (mean 59.1 ± 12.0│ median 60, interquartile range (IQR) 13.7 for females; mean 59.7 ± 10.5│median 58.5, IQR 10.7 for males; P = 0.816). As for education, 56.7% (n = 59) of the individuals were literate, with no difference between the sexes (χ² test; P = 0.319), although 59.3% (n = 35) were female. The illiterate were older (P < 0.001) (Mean 65.4 ± 9.3│median 64, IQR 11) when compared to the literate (57.0 ± 10.6│median 57, IQR 12). In addition, the mean number of years of schooling was 7.9 ± 4.3│median 7.5, IQR 6.0), with no difference between the sexes (P = 0.614) (mean 7.7 ± 4.5│median 7.0, IQR 7 for females; mean 8.1 ± 4.1│median 8, IQR 6 for males) (Table 1a).

**Table 1:** Characterization of life habits and comorbidities of the Fulni-ô population. Águas Belas, Pernambuco, Brazil (n = 104)

**a) Sociodemographic variable**		Total (n = 104)	Female (n = 66)	Male (n = 38)
Age - median (IQR)		59.0 (11.0)	60.0 (13.7)	58.5 (10.7)
Literate (n; %)		59 (56.7%)	35 (59.3%)	24 (63.2%)
Years of schooling - median (IQR)		7.5 (6.0)	7.0 (7.0)	8.0 (6.0)
**b) Lifestyle (categorical variables)**		**n (%)**	**n (%)**	**n (%)**
**Alcoholic beverage (n = 87)**				
	Never	61 (70.1%)	49 (87.5%)	12 (38.7%)
	Active	8 (9.2%)	4 (7.1%)	4 (12.9%)
	Stopped	18 (20.7%)	3 (5.4%)	15 (48.4%)
**Do you use any tobacco products? (n = 100)**				
	Yes	94 (94.0%)	59 (93.7%)	35 (94.6%)
	No	6 (6.0%)	4 (6.3%)	2 (5.4%)
**Do you use commercial cigarettes? (n = 53)**				
	Yes	14 (26.4%)	12 (38.7%)	2 (9.1%)
	No	39 (73.6%)	19 (61.3%)	20 (90.9%)
	Cigarette (Average daily consumption - units)^1^	6.5 ± 7.8	7.18 ± 8.4	3.0 ± 0.0
**Do you use traditional Xanduca pipe? (n = 57)**				
	Yes	4 (7.0%)	3 (9.7%)	1 (3.8%)
	No	53 (93.0%)	28 (90.3%)	25 (96.2%)
**Do you chew smoke? (n = 47)**				
	Yes	1 (2.1%)	1 (3.4%)	0 (0.0%)
	No	46 (97.9%)	28 (96.6%)	18 (100.0%)
**Inhaled smoke (n = 47)**				
	Yes	2 (4.2%)	1 (3.5%)	1 (5.5%)
	No	45 (95.8%)	28 (96.5%)	17 (94.5%)
**Cardiovascular complaints? (n = 101)** 2				
	Yes	26 (25.7%)	14 (22.2%)	12 (31.6%)
	No	75 (74.3%)	49 (77.8%)	26 (68.4%)
**Comorbidities (n = 43)**				
	Stroke	2 (4.7%)	0 (0.0%)	0 (0.0)
	Dyslipidemia	6 (14.0%)	5 (23.1%)	1 (6.3%)
	Diabetes	3 (7.0%)	3 (11.5%)	0 (0.0%)
	Systemic arterial hypertension	32 (74.3%)	17 (65.4%)	15 (93.8%)
**c) Traditional pipe (continuous variables)**				
**Variable**		Mean ± SD (Median; IQR)	Mean ± SD (Median; IQR)	Mean ± SD (Median; IQR)
Xanduca (average daily consumption - units)^2^		4.5 ±5.2 (3.0; 3.0)	4.2 ±4.0 (3.0; 3.0)	4.9 ±6.7 (3.0; 2.0)
Consumption time (in years)^3^		43 ±39.2 (40.0; 15.0)	44 ±48.9 (38.0; 16.0)	39.7 ±40.7 (40.0; 12.7)
Consumption load (years/Xanduca)^4^		188.2 ±251.9 (110.5; 124.7)	182.5 ±236.3 (110.5; 131.2)	198.3 ±280.1 (114.0; 121.2)

SD = standard deviation; IQR = interquartile range.

1 χ^2^ tests (P = 0.297);

2 U-Mann-Whitney test (P = 0.579);

3 U-Mann-Whitney test (P = 0.591);

4 U-Mann-Whitney test (P = 0.519). Cardiovascular complaints included chest pain, dyspnea, and claudication.

Alcohol consumption (current and past) was observed in 29.9% of the individuals and was more prevalent in the male population (61.3% of men consume or have consumed alcohol regularly). The prevalence of tobacco derivative use was 94.0%, with similarities between the sexes. However, the percentage of cigarettes smoked was 4.2 times higher in the female population, as well as in Xanduca with herbs (2.5 times). The frequency of cardiovascular complaints was 25.7%, and was higher in the male population (31.6%), although the difference was not significant (P = 0.297) (Table 1b).

The average daily consumption of Xanduca was 4.5 ± 5.2 pipes (median, 3.0; IQR 3.0), with no difference between the sexes (P = 0.579). The mean time of smoking was 43 ± 39.2 years (median 40.0; IQR 15.0), with no difference between the sexes (P = 0.591). The same was also observed for the consumption of Xanduca: mean of 188.2 ± 251.9 years/Xanduca (median 110.5; IQR 124.7), with no difference between the sexes (P = 0.519) (Table 1c).

The prevalence of oral alterations was 84.4% (n = 92), including halitosis at 86.5% (n = 90), caries at 77.9% (n = 81), extrinsic dental pigmentation at 75.0% (n = 78), periodontitis at 73.1% (n = 76), and gingivitis at 71.2% (n = 74). Eight lesions were prevalent in females (oral candidiasis, halitosis, periodontitis, gingivitis, extrinsic dental pigmentation, papilloma, caries, and occlusal wear). Furthermore, no difference was observed between sexes in lesions with malignant potential (actinic cheilitis, leukoplakia, and erythroplakia) (Table 2).

**Table 2:** Prevalence of oral cavity involvement observed in the Fulni-ô population. Águas Belas, Pernambuco, Brazil (n = 104)

**a) Prevalence of oral lesions**				
**Characteristics**	Total (n = 104) n (%)	Female (n = 66) n (%)	Male (n = 38) n (%)	χ^2^ test (P value)
Oral candidiasis	19 (18.3)	14 (73.6)	5 (26.4)	0.039*
Halitosis	90 (86.5)	56 (56.0)	34 (34.0)	0.020*
Periodontitis	76 (73.1)	48 (63.2)	28 (36.8)	0.022*
Gingivitis	74 (71.2)	46 (62.2)	28 (37.8)	0.036*
Extrinsic tooth pigmentation (smoking)	78 (75.0)	49 (62.8)	29 (37.2)	0.024*
Papilloma (HPV)	8 (7.7)	7 (87.5)	1 (12.5)	0.034*
Caries	81 (77.9)	52 (64.2)	29 (35.8)	0.011*
Hemangioma	6 (5.8)	5 (83.3)	1 (16.7)	0.102
Nicotinic stomatitis (hard palate)	27 (26.0)	19 (70.4)	8 (29.6)	0.304
Reactional/inflammatory hyperplasia	56 (53.8)	37 (66.1)	19 (33.9)	0.481
Actinic cheilitis	12 (11.5)	9 (75.0)	3 (25.0)	0.083
Leukoplakia	25 (24.0)	13 (52.0)	12 (48.0)	0.841
Erythroplakia	17 (16.3)	12 (70.6)	5 (29.4)	0.090
Occlusal wear	4 (3.8)	4 (100.0)	0 (0.0)	0.046*
**b) Diagnosis description obtained from tissue biopsy (n = 18** 1 **)**				
**Characteristics**	**n**	**Sex**	**Age**	**Consumption of Tobacco derivatives or Xanduca with Herbs - in years (CL)**
Mild oral epithelial dysplasia	5	3M│2F	66**	47 (218)**
Acanthosis	4	3M│1F	64**	47 (455)**
Moderate dysplasia	1	M	72	52 (52)
Nonspecific inflammatory infiltrates	2	2F	63│62	46 (184)│46 (276)
Inflammatory fibrous hyperplasia	2	M│M	84│71	62 (248)│55(1100)
Hyperkeratosis	1	M	60	40 (160)
Giant Cell Fibroma	1	F	46	22 (88)
Solar elastosis	2	M│F	84│63	62 (240)│46(184)
**Total**	**18**			

CL = consumption load; F = female; M = male.

In total,

1 15 samples were collected from 13 patients. The sum of 18 in this table indicates that an individual had more than one diagnostic finding.

* Statistical significance (P <0.05)

** Group Average.

**Figure 2:**
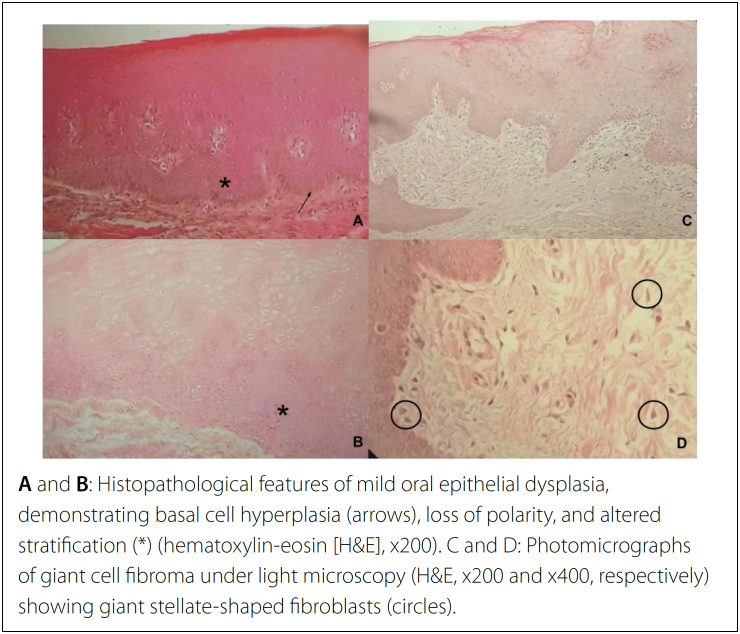
Histopathological aspects observed in patients undergoing biopsy.

Fifty-one people who underwent oral reassessment were referred for oral lesion biopsy. However, only 13 agreed to participate (Table 3). Fifteen biopsy fragments were collected from those who agreed. Histopathological analyses showed eight different oral pathologies, with emphasis on five cases of mild oral epithelial dysplasia; four lesions were brown-to-black, poorly defined, with velvety hyperpigmentation of the skin, and classified as acanthosis (Table 3 and Figure 2).

**Table 3:** Fulni-ô individuals at the time of collection of material for biopsy. Águas Belas, Pernambuco, Brazil (n = 51 individuals with lesions suggestive of malignancy)

Description	n
Patients with biopsies performed	**13**
Samples collected at the time	**15**
Individuals with characteristic precancerous lesions. Clinical examination and testing ruled out fungal lesions. They refused to perform biopsy	**06**
Individuals who refused	**10**
Individuals not found	**06**
Individual detained in a prison unit	**01**
Individuals who have moved out of state	**02**
Death from cardiovascular disease	**01**
Individuals traveling on the day of collection	**02**
Individuals without suggestive lesions at the time of collection. Other diagnoses were observed: fungal diseases, hyperplasia due to ill-fitting prostheses, and unfavorable hygienic conditions	**12**

## DISCUSSION

It is essential that periodic surveys be part of a strategy incorporated in Indigenous Health Policy from the perspective of building a historical series of oral health data capable of subsidizing the development of plans and public policies. The present investigation showed a high prevalence of oral impairment in the Fulni-ô indigenous population. This is the first study to be carried out in this population.

The National Oral Health Policy (Smiling Brazil)^17^ has its main health surveillance mechanism in national surveys, such as Oral Health Brazil 2003 and Oral Health Brazil 2010. Based on them, in December 2011, the Ministry of Health began the process of implementing Smiling Indigenous Brazil, a branch of the PNSB focused exclusively on the oral health needs of indigenous populations.^18,19^ Although it was an important step, at that moment, only the Special Indigenous Health Districts (DSEIs) of Alto Rio Solimões (Amazonas), Alto Rio Purus (Acre), and Xavante (Mato Grosso) were contemplated.^18,19^

In subsequent years, these actions expanded to other DSEIs. Between 2014 and 2018, the number of consultations provided by dentists increased slightly, from 29,000 to more than 177,000. The number of consultations conducted by oral health technicians or assistants increased from slightly from over 11 thousand in 2014 to almost 160 thousand in 2018. Another important aspect of the 2018 survey was the performance of Indigenous professionals in the care of their population (of the 450 oral health technicians working in 2018, 250 were indigenous).^20^

However, the oral healthcare offered varies among the states and regions of Brazil. While the coverage of dental consultations grew by 39.23% in the state of Ceará and 18.47% in Bahia, Pernambuco, where the Fulni-ô ethnic group is located, a reduction of 2.75% was observed.^20^ These inequalities generate important losses for the oral health of the population given the interruption of longitudinal care, a fundamental element for the construction of sustainable health practices.

This context becomes even more relevant when we consider the sociocultural and historical aspects of the indigenous Brazilians. Indigenous knowledge about health is based on their own methods of interpretation, prevention, treatment, and cure of pathologies, which are associated with sociocultural, historical and environmental factors.^21–23^ Therefore, health and disease are extremely complex processes because they combine biological, environmental, socioeconomic, and cultural factors, which makes it impossible to establish a hierarchy among them.

Regarding the oral health conditions of indigenous people, there is an association between the deterioration of oral health and the consumption of industrialized food, involving the precariousness of dental care.^24,25^ There are signs that the increased prevalence of caries in indigenous populations can be attributed to changes in diet, combined with socioeconomic and environmental changes and lack of programs.^24^ Since the 1990s, epidemiological transition and cross-cultivation had already been identified in the emergence of diseases in general and of dental abscesses.^26^

In this perspective, high rates of dental caries are noted, as shown by some epidemiological studies conducted on indigenous populations, such as the *Sateré-Mawé* and *Tikuna* peoples of the upper Negro River, Amazonas,^27^ *Kaingang* from Rio Grande do Sul.^28^ In general, this phenomenon is repeated throughout the Brazilian territory.^29^ Despite the high smoking tobacco usage, potentially malignant disorders of the oral mucosa were uncommon when compared with ordinary Brazilian populations,^30^ and no malignant neoplasia was found.

The profile of individuals diagnosed with a potentially malignant lesion in our study, males in their 50s or older and those who use tobacco are in agreement with what has been previously described in the literature,^31^ but the absence of malignant lesions in the oral cavity of these individuals needs further evaluation. In a study of indigenous people of the Guarani Kaiowá/Nandeva ethnic group from Mato Grosso do Sul, 406 pathological alterations were observed, although only 14.4% were lesions, including leukoplakia, nicotinic estimatitis, fibroma, and ulceration.^31^

Fibrous inflammatory hyperplasia is a reactive lesion related to trauma of oral mucosa and with a great prognosis and no malignant-related transformation.^32^ Giant cell fibroma is a benign lesion of the oral cavity with distinctive etiopathology different from traumatic oral lesions, which is predominantly found in Caucasians and rarely in other races.^33^ It remains unclear if a viral infection precedes its proliferative nature, but etiology is unclear.^34^ Finally, solar elastosis is a skin damage of the lip caused by ultraviolet exposures, which may be histologically associated with epithelial dysplasia and considered a potentially malignant lesion. Fortunately, this condition can be stabilized or reversed with proper treatment.^35^

Tobacco use kills more than 8 million people each year.^36^ It is considered to be the largest preventable cause of illness and early death worldwide.^37^ In fact, smoking is a major risk factor for the development of several types of cancer,^38^ including oral cancer. However, little is known about the effects of traditional pipe use on the health of populations, particularly indigenous people. In Brazil, the native population has a habit of smoking a traditional pipe in purification rituals and approximating its divinities, maintaining the link between individuals and their spirituality.^13^

Smoking a traditional pipe (Xanduca) has a high prevalence in the Fulni-ô indigenous community.^13^ The use of pipes and malignant lesions in the oral mucosa has been shown for the general population.^39^ However, the extent of the pipe-related damage to the oral health of indigenous people is still unkown.

Even considering the methodological precautions, this study has limitations, among which we highlight as follows: i. the concomitant use of traditional pipes with herbs and tobacco is a confounding factor of the study; ii. a case-control study could provide more solid evidence on the effects of traditional pipes compared with the use of tobacco; iii. memory bias may have influenced the answers, especially regarding the time of consumption; iv. Oral hygiene habits were not assessed in the study; v. type of sample adopted (non-probabilistic); and vi. the small sample size, with a predominance of women, made it difficult to reliably assess injuries between men and women.

## CONCLUSIONS

This study showed a high prevalence of oral alterations in the Fulni-ô population, especially halitosis, caries, extrinsic dental pigmentation, periodontitis, and gingivitis. Histopathological analyses showed eight different oral pathologies, with emphasis on five cases of mild oral epithelial dysplasia; four lesions were brown-to-black, poorly defined, with velvety hyperpigmentation of the skin, and classified as acanthosis.

Further studies should be conducted in this population to characterize oral hygiene habits and understand the influence of traditional pipes on oral health.
